# Melatonin: shedding light on infertility? - a review of the recent literature

**DOI:** 10.1186/s13048-014-0098-y

**Published:** 2014-10-21

**Authors:** Shavi Fernando, Luk Rombauts

**Affiliations:** MIMR-PHI Institute of Medical Research, 246 Clayton Rd, Clayton, 3168 Victoria Australia; Monash University, Department of Obstetrics and Gynaecology, Level 5 Monash Medical Centre, 246 Clayton Rd, Clayton, 3168 Victoria Australia; Monash IVF, 252 Clayton rd, Clayton, 3168 Victoria, Australia

**Keywords:** Melatonin, Oxidative stress, Oxygen scavenger, Infertility, In-vitro fertilisation

## Abstract

In recent years, the negative impact of oxidative stress on fertility has become widely recognised. Several studies have demonstrated its negative effect on the number and quality of retrieved oocytes and embryos following in-vitro fertilisation (IVF). Melatonin, a pineal hormone that regulates circadian rhythms, has also been shown to exhibit unique oxygen scavenging abilities. Some studies have suggested a role for melatonin in gamete biology. Clinical studies also suggest that melatonin supplementation in IVF may lead to better pregnancy rates. Here we present a critical review and summary of the current literature and provide suggestions for future well designed clinical trials.

**Electronic supplementary material:**

The online version of this article (doi:10.1186/s13048-014-0098-y) contains supplementary material, which is available to authorized users.

## Introduction

Over the last 35 years, infertility treatment has become more acceptable and with improvements in technology the pressure for improved success rates has mounted. This trend is perpetuated by a perceived ability to delay and yet successfully achieve pregnancy through assisted reproductive technologies (ART) [1]. Technological advancement and societal expectations therefore mandate continual improvement in in-vitro fertilisation (IVF) success rates, inspiring research into novel adjuvant therapies designed to improve IVF outcomes. More recently, it has been discovered that an imbalance of reactive oxygen species, or `oxidative stress', can have a negative impact on the success of infertility treatments, and furthermore, investigators have begun addressing potential mechanisms of preventing these effects with the use of novel oxygen scavengers such as melatonin. It may be that these agents have a positive effect on pregnancy success rates following IVF treatment. We present a summary of the most recent work investigating melatonin and its affect on oxidative stress, with a focus on the reproductive system and the treatment of infertility.

## Melatonin

### Melatonin: synthesis and degradation

Melatonin (N-acetyl-5-methoxytryptamine) was first isolated in 1958 as a neuro-hormone mainly synthesised and secreted from the pineal gland [2]. Since its discovery, further investigation has revealed that it is also produced by several other organs. It has been found in the gastrointestinal tract [3], brain [4], eye [5], lungs [6], skin [7], kidney [8], liver [9], thyroid, thymus, pancreas [10], immune system [11] and reproductive system [12]. Melatonin is an indoleamine, which is synthesised from the essential amino acid, tryptophan [13]. Its production is dependent on ambient illumination, with release being suppressed by light. Hence, endogenous levels in plasma begin to increase between 1800 and 2000 hrs and peak between midnight and 0500 hrs with levels before 0900 hrs being five times higher than those after 1100 hrs [14]. This diurnal variation can make comparative studies challenging.

In an investigation of the pharmacokinetics of exogenous orally administered melatonin, Waldhauser and associates found that the increase in serum levels after oral administration of melatonin is rapid (60–150 minutes), as is its excretion [15]. It does not accumulate in the blood, with repeat dosing simply resulting in peak levels being maintained for longer [15]. Melatonin is hepatically metabolised and renally excreted [16]. Hence, melatonin has a short half-life and both melatonin and its metabolites can be measured in serum, urine and saliva [17],[18].

### Actions and safety of melatonin

#### Classical actions

Melatonin has been identified as a key factor in the regulation of circadian rhythms and the sleep-wake cycle [18]. Long exposure to artificial lighting leads to a reduction in endogenous melatonin exposure [19]. Melatonin is thus associated with sleep disturbances including insomnia, and much of the literature is focused in this area [20]-[22]. It also appears to regulate reproductive seasonal variation in many animal species [18],[23]-[25]. However, despite a daily circadian rhythm being demonstrated in uterine artery blood flow [26], seasonal breeding does not apply to primates [27], raising questions as to what other roles it may serve in humans.

#### Actions as an oxygen scavenger

Free oxygen radicals are created when oxygen is utilised in metabolic processes. These radicals contain `free’ valence electrons, making them highly reactive, capable of causing injury to cells [28]. The term `reactive oxygen species' (ROS), not only includes free radicals but also stable non-radical molecules which are capable of causing oxidation, such as hydrogen peroxide (H_2_O_2_) [29]. While ROS are necessary for essential physiological processes, an overabundance can result in cellular damage, commonly referred to as `oxidative stress´ [30]. Anti-oxidative agents (oxygen-scavengers) are present endogenously but can also be administered exogenously. They reduce free radicals by donating electrons to stabilise them [31].

**Figure 1:**
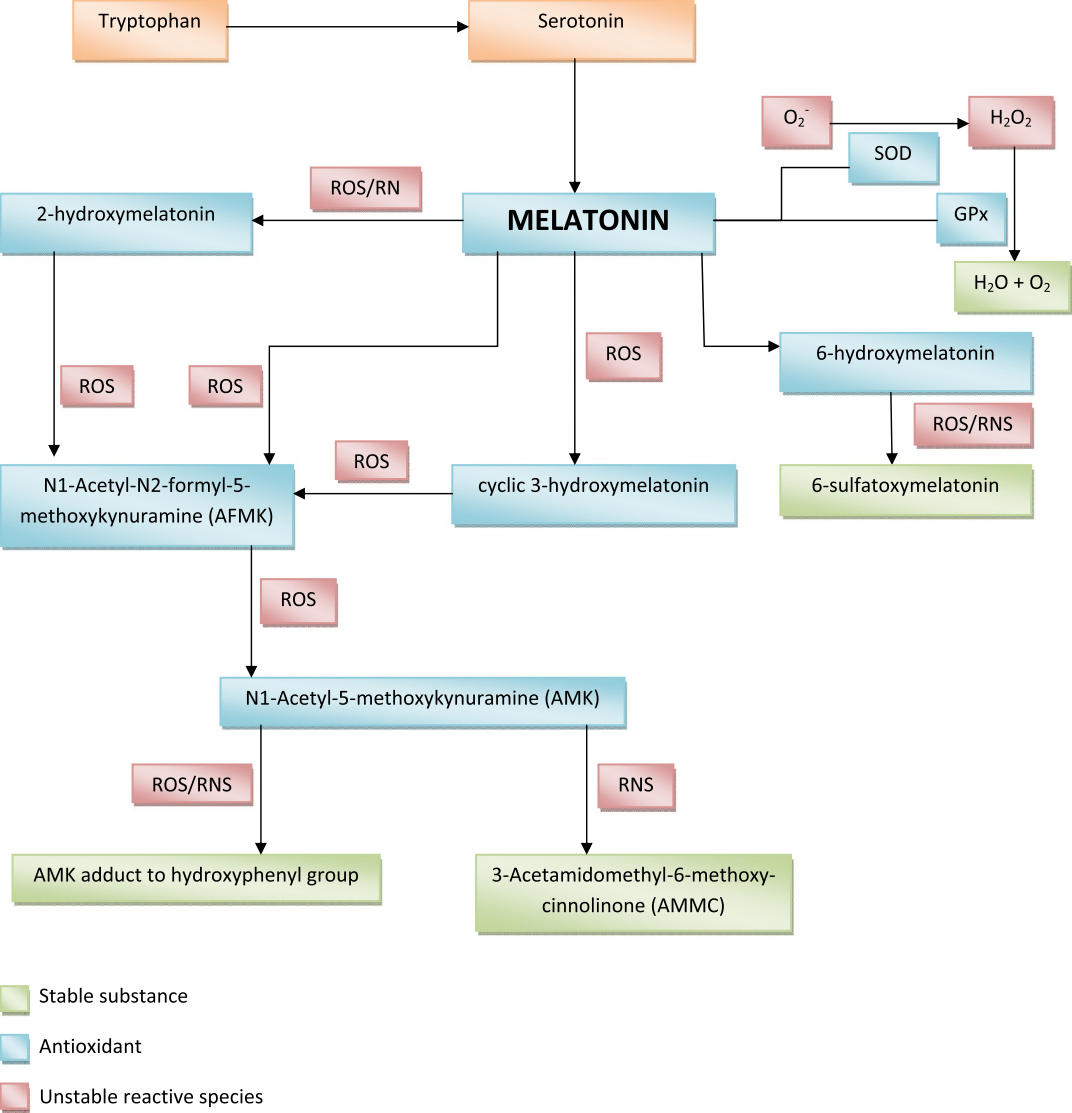
**Actions of melatonin and its metabolites.** Extrapolated from Hardeland [46], Reiter et al. [13] and Watson [47]. GPx: glutathione peroxidase; SOD: superoxide dismutase; ROS: Reactive oxygen species; RNS: Reactive nitrogen species.

Recently, it has been discovered that melatonin has important oxygen-scavenging properties [32]-[34]. Compared with other oxygen scavengers, melatonin is of particular interest because it has several qualities distinguishing and rendering it superior to classical anti-oxidative agents. For example, it has anti-oxidative effects through its receptors, MT1 and MT2 [35], but also as a direct free radical scavenger [36],[37]. It has binding sites within the nucleus [13],[38], and is amphiphilic, allowing it to cross cell membranes freely [36],[39]. But one of its most unique characteristics is that, unlike classical anti-oxidants, melatonin is a suicidal terminal anti-oxidant. It does not promote oxidation under any circumstances and its metabolites are also capable of acting as anti-oxidants in a `scavenging cascade reaction´ without themselves becoming oxidative [37],[40]–[42]. Importantly, melatonin also enhances the activity of other endogenous anti-oxidants including glutathione peroxidase and superoxide dismutase (Figure 1) [43]–[47].

These unique characteristics have made melatonin the subject of investigation into medical conditions in which oxidative stress has been implicated, including diabetes, glaucoma, irritable bowel syndrome and even in curtailing the side effects of chemo- and radiotherapy [48],[49]. Melatonin has been shown to suppress tumour growth factors and angiogenesis, suggesting a possible role for melatonin in prevention of cancer growth [9],[50],[51]. Furthermore, melatonin has been shown to have anti-inflammatory and DNA stabilising actions in the lung [6],[52], skin and intestine [53]–[56] and can help reduce chronic pelvic pain in women with endometriosis [57].

#### Importance of melatonin in reproduction

**Figure 2:**
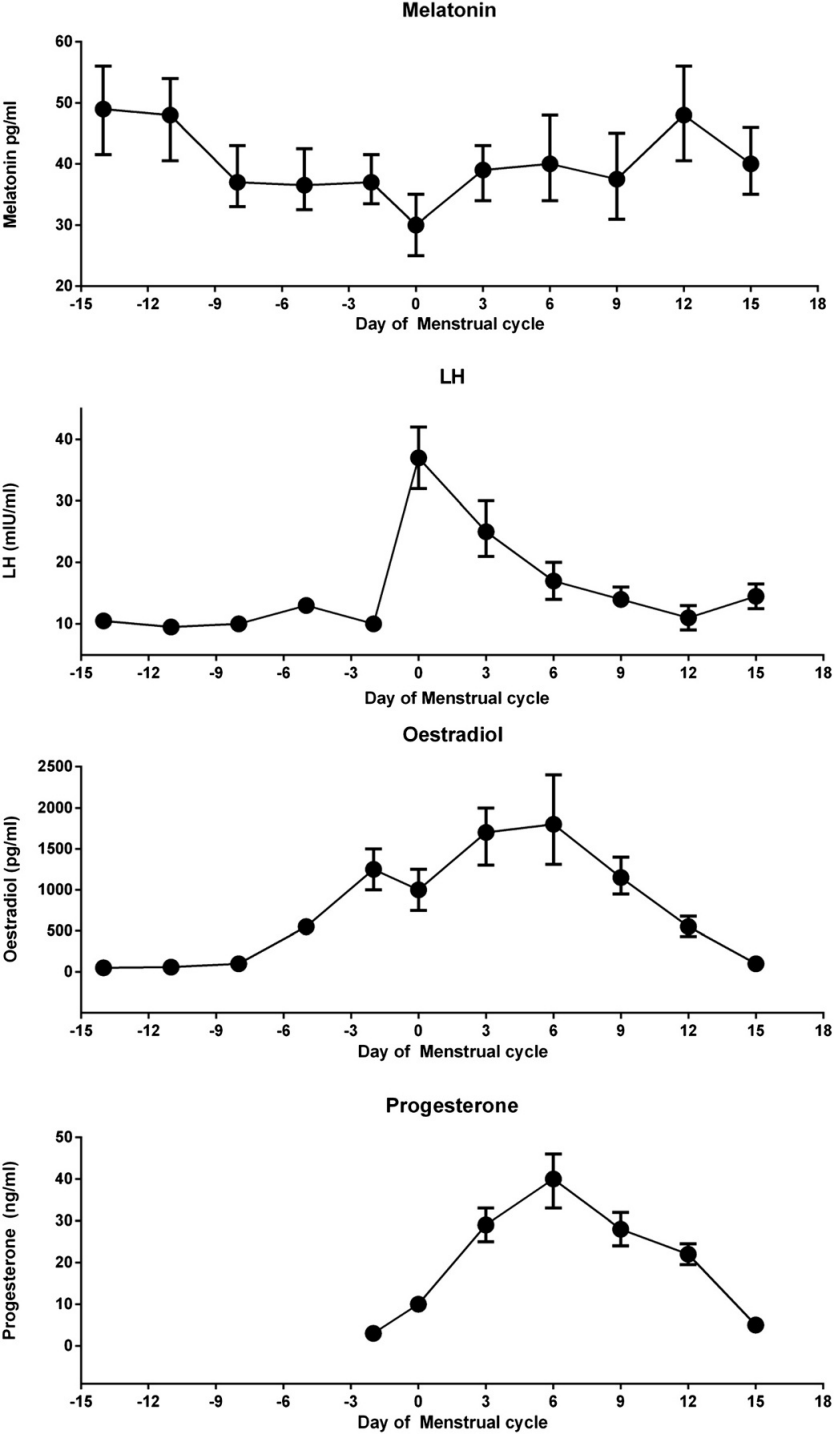
**Relative concentrations of plasma melatonin, LH, estradiol and progesterone in hMG/hCG treated cycles.** Adapted with permission from Tang et al. [59]. LH: Luteinising hormone.

In humans, the only data on cyclical melatonin changes comes from women undergoing ovarian stimulation. Levels of melatonin reach a nadir in the preovulatory phase and peak in the luteal phase (Figure 2) [58]–[60]. This suggests that melatonin has variable effects dependent on the menstrual phase.

It is also well known that shift-workers are more likely than daytime workers to experience circadian disruption and longer menstrual cycles, more menorrhagia and dysmenorrhoea [61],[62]. These results are corroborated by a very large cohort study, which also found that duration of shiftwork was modestly associated with menstrual cycle irregularity [63]. A Japanese study found that melatonin levels varied significantly between night and day shift workers, while LH and FSH levels did not, suggesting that the menstrual irregularity associated with shift-work could be explained by melatonin fluctuations [64].

These findings are in line with central effects on the hypothalamic pituitary axis, being capable of modifying the release of gonadotrophins and GnRH [65]. In fact, in very high doses, when combined with progesterone, melatonin has the ability to suppress ovulation in humans, possibly by interfering with LH release [66]. This may represent an evolutionary remnant with inhibition of ovulation during darker months designed to prevent the birth of offspring when resources are less abundant.

Interestingly, melatonin receptors have been found on granulosa cells, indicating that this may be an additional site of melatonin activity [65],[67],[68]. Indeed, when given systemically in cats, melatonin appears to accumulate preferentially in the ovaries compared with other organs [69] and higher concentrations of melatonin are found in preovulatory follicular fluid than in serum [36],[70],[71]. A human study by Nakamura et al. [72] found that larger preovulatory follicles had higher concentrations of follicular fluid melatonin than smaller immature follicles. This is the only study that has addressed follicular fluid differences within the same patient, and indicates that follicular fluid from mature follicles have higher anti-oxidant capacity than smaller follicles, implying a role for melatonin in oocyte maturation. However, it is as yet unclear whether this is a cause or consequence.

Adding further credence to the role of melatonin in reproduction, melatonin requirements appear to increase during pregnancy [73], and researchers have begun to assess its role as a potential therapy in pre-eclampsia and neonatal neurological morbidity [74],[75]. Recent investigations have shown that in ovine models, intrauterine infusion of melatonin results in an increase in umbilical artery blood flow and higher fetal-placental weight ratio. Importantly, intrauterine infusion of a melatonin receptor antagonist decreased fetal aortic blood flow relative to fetal weight, suggesting that activation of melatonin receptors may be the mechanism behind the apparent increase in fetal blood flow after oral melatonin supplementation [76]. Melatonin has also been shown to reduce the neurological effects of oxidative stress-induced fetal brain injury in rats and sheep [77],[78]. These findings support a beneficial role of melatonin in the treatment and/or prevention of placental dysfunction, which may even extend to the treatment of pre-eclampsia and neurological damage in preterm and growth restricted neonates [4],[78].

Because melatonin levels naturally decrease with age [79],[80], some investigators have found that supplementation may also have a role in the climacteric [81]. Melatonin also appears to have a role in the prevention of postmenopausal bone loss, with effects being exerted via inhibition of oxidative stress, induction of osteoblastogenesis and inhibition of osteoclastogenesis [82]. These findings and evidence from a small randomised controlled trial suggests that melatonin may be useful in the treatment of perimenopausal and menopausal symptoms and sequelae [83],[84].

The positive implications of higher melatonin levels on the human menstrual cycle, fertility and pregnancy are therefore well documented, with varying levels of evidence [32]–[34],[85]. Nevertheless, it appears clear that melatonin serves a purpose in the human reproductive system, with many of its observed effects likely to be related to its ability to dampen the effects of oxidative stress on the reproductive system.

#### Safety

Given the potential clinical benefits of melatonin it is equally important to assess its potential for harm, particularly when considering treatment in infertile or pregnant populations. It is reassuring that melatonin has a remarkably benign safety profile in both animal and human studies, with no teratogenic effects [86]–[88]. Furthermore, melatonin does not have significant sedative effects and is not associated with hepato-nephrotoxicity [89] even at supraphysiological doses (5 - 20 mg/day) for prolonged periods of administration (up to 12 weeks) in both adults and children [66],[90]–[92]. While non-toxic, it has been suggested that melatonin can adversely affect autoimmune conditions, particularly rheumatoid arthritis [93], through its immuno-stimulatory actions. There have also been two case-reports of melatonin being associated with autoimmune hepatitis [94],[95], and a suggestion that it may be implicated in multiple sclerosis through T-cell activation [96]. Though these effects are associative and follow biological plausibility, causality has not yet been proven [97]. Despite this, it is recommended to avoid the use of melatonin in those with autoimmune conditions.

## Infertility treatment

### The importance of oxidative stress in assisted reproductive technology (ART)

The relevance of oxidative stress in ART has gained increasing attention in recent literature, in particular with regards to IVF. IVF can result in exposure of oocytes and embryos to high levels of superoxide free radicals, which begins prior to oocyte retrieval [98]. Ovarian stimulation protocols are associated with significant changes to the *in-vivo* follicular environment, altering endogenous levels of oxygen scavengers [99]. Furthermore, in-vitro, these oocytes are no longer protected by antioxidant-rich follicular fluid, leaving them more susceptible to oxidative damage [100]–[102]. They may also be exposed to high oxygen concentrations in incubators and during handling throughout the IVF process, with higher concentrations of oxygen being associated with more ROS, and a positive effect of melatonin being more marked in oocytes exposed to higher oxygen tensions [103]. This oxidative stress modifies the quality of oocytes and embryos, decreasing the fertilisation rate and the success of the infertility treatment [104]–[106].

Investigators have found an inverse relationship between follicular fluid levels of ROS and success of ART, and these differences do not seem to be related to the cause of infertility. Bedaiwy et al. sequentially analysed the follicular fluid from 138 patients undergoing intra-cytoplasmic sperm injection ICSI [107]. They found that cycles that resulted in pregnancy were associated with a significantly higher total antioxidant capacity (a measure of the summative effect of antioxidants in the serum [108]) and significantly lower level of ROS [107] but the sample sizes were relatively small. This evidence suggests that intra-follicular oxidative stress may have a significant impact on IVF success rates.

While reactive oxygen species are required for sperm capacitation events [109],[110], an imbalance of ROS has been implicated as a factor in reducing the quality and function of sperm [111], with most protection from these effects being afforded by the enzymatic antioxidant, superoxide dismutase [112]–[116].

One reason for the increased susceptibility of sperm to oxidative stress is the abundance of oxidative targets such as polyunsaturated fatty acids in the plasma membrane of sperm required for fusion with the oocyte and fertilisation [117]. Furthermore, it has been found that DNA fragmentation resulting from both *in-vivo* and in-vitro oxidative stress is a major contributor to poor sperm quality and function, and that antioxidative therapies may hold promise in attenuating these effects [118]. As might be expected, such DNA damage has been shown to have a negative impact on fertilisation, blastocyst development [119], and miscarriage rates and pregnancy outcome [120].

The recognition of the association between exposure of gametes and embryos to oxidative stress and a reduction in the success rates of IVF has led investigators to assess whether these adverse effects can either be prevented or reversed, with emphasis being placed on the adjuvant use of oxygen scavengers including melatonin.

### The role of melatonin in assisted reproductive technology

Oxidative stress occurs at many levels during the treatment of infertility. Interventional studies have begun recently, with an emphasis on oral supplementation of melatonin during the ovarian stimulation phase of the IVF cycle and its effects on gamete and embryo quality. Clinical studies assessing the use of melatonin in IVF are summarised in Table 1 and discussed in more detail below.

**Table 1:** **Summary of human studies assessing the use of melatonin in IVF**

Study	Design	NICE Level of evidence	Sample size	Intervention	Control	Outcomes
**Melatonin alone**						
Tamura et al. 2012 [36]	Uncontrolled before - after study	2^–^	9	3 mg melatonin po from day 5 of menstrual cycle to oocyte collection (n = 9)	Previous cycle without melatonin (n = 9)	Higher rate of good embryos in melatonin cycle (65% vs 27%)*
Tamura et al. 2008 [125]	Prospective cohort	2^+^	115	3 mg melatonin po from day 5 to oocyte collection (n = 56)	No melatonin (n = 59)	No difference in fertilisation or clinical pregnancy rate
Tamura et al. 2008 [125]	Uncontrolled before - after study	2^–^	112	3 mg melatonin po from day 5 to oocyte collection (n = 56)	Previous cycle without melatonin (n = 56)	Higher fertilisation rate in melatonin cycle (50% vs 20.2%)*
						No difference in pregnancy rate
Eryilmaz et al. 2011 [137]	Unblinded randomised controlled trial	1^–^	60	3 mg melatonin po from day 3–5 until HCG injection (n = 30)	No melatonin (n = 30)	Higher number of oocytes in melatonin group (11.5 vs 6.9)*
						Higher MII oocyte counts (9 vs 4.4)*
						Higher G1 embryo transfer rate (69.3 vs 44.8)*
						No differences in fertilisation, implantation or clinical pregnancy rates
Batioglu et al. 2012 [138]	Single-blinded randomised controlled trial (only embryologists were blinded)	1^–^	85	3 mg melatonin po (n = 40)	No melatonin (n = 45)	Higher percentage of MII oocytes in melatonin group (81.9% vs 75.8%)*
						Higher number of G1 embryos (3.2 vs 2.5)*
						No difference in number of oocytes, fertilisation rate or clinical pregnancy rate
Nishihara et al. 2014 [134]	Uncontrolled before - after study	2^–^	97	3 mg melatonin po for at least 2 weeks leading up to HCG trigger in second cycle (n = 97)	No melatonin in first cycle (n = 97)	Higher ICSI fertilisation rate in melatonin group (77.5% vs 69.3%)*
						Higher rate of good quality embryos (Day 3) (65.6% vs 48.0%)*
						No difference in maturation rate, blastocyst rate or good quality blastocysts (Day 5)
**Combinations with melatonin**						
Rizzo et al. 2010 [139]	Unblinded randomised controlled trial	1^–^	65	3 mg melatonin daily +2 g myo-inositol po bd +200mcg folic acid po bd from day of GnRH administration (n = 32)	2 g myo-inositol po bd +200mcg folic acid po bd from day of GnRH administration (n = 33)	Higher number of MII oocytes in melatonin group (6.56 vs 5.76)*
						Lower number of immature oocytes (1.31 in vs 1.90)*
						No difference in fertilisation rate, embryos transferred, implantation rate or clinical pregnancy rate
Unfer et al. 2011 [165]	Uncontrolled before - after study	2^–^	46	2 g myo-inositol po +200mcg folic acid po in the morning and 3 mg melatonin po +2 g myo-inositol po +200mcg folic acid po in the evening for 3 months leading to second cycle of IVF	No trial medication in first cycle	Higher number of MI and MII oocytes in treatment cycle (3.11 vs 2.35)*
						Higher number of G1 or G2 embryos transferred (0.35 vs 0.13)*
						Clinical pregnancy rate 19.6% in treatment cycle
						No differences in number of oocytes or fertilisation rate
Pacchiarotti et al. 2013 [164]	Double-blinded randomised controlled trial	1^+^	388	3 mg melatonin po +4 g myo-inositol po +400mcg folic acid po (n = 178)	4 g myo-inositol +400mcg folic acid po (n = 180)	Higher percentage of mature oocytes in melatonin group (48.2% vs 35.0%)*
						Higher percentage of G1 embryos (45.7% vs 30.4%)*

*IVF*: In-vitro fertilisation; *NICE*: National Institute for Health and Care Excellence; *statistically significant; *G1*: Grade 1; *G2*: Grade 2; *MI*: Meiosis I; *MII*: Meiosis II; *ICSI*: Intracytoplasmic sperm injection; *HCG*: Human chorionic gonadotrophin; *po*: per oral; *bd*: Twice per day.

#### Effects of melatonin on oocyte quality

Melatonin is an effective mitigator of mitochondrial DNA damage [121], likely as a result of an increase in electron transport efficiency within mitochondria, thus preventing the formation of ROS [122]. In some situations melatonin may be even more effective at performing this function than specific mitochondrial antioxidants [123], and this particular characteristic may have relevance to its use in the treatment of infertility and the improvement of oocyte quality and maturity.

Oocyte quality begins to deteriorate immediately following ovulation, a process thought to be inflammatory [124] and through its production of cytokines and proteases is associated with an increase in ROS which can inhibit oocyte maturation [13],[125],[126]. A very recent murine study found that oxidative stress in oocytes may begin as early as 8 hours after ovulation, rising exponentially thereafter. This study also found that in-vitro addition of 1 mM of melatonin to oocyte culture media significantly ameliorated these time-dependent effects, resulting in 54% of fertilise oocytes reaching the blastocyst stage in the presence of melatonin compared with 29% in the controls [127]. This study not only showed that an imbalance of ROS is an important cause of impaired oocyte quality in-vitro, but also that the addition of melatonin could reverse these effects.

The follicular environment is naturally protective against oxidative damage to the oocyte [128]. To illustrate this, Tamura et al. sampled follicular fluid at oocyte retrieval and measured intrafollicular concentrations of melatonin and the oxidative stress marker, 8-hydroxy- 2'-deoxyguanosine (8-OHdG). Melatonin concentrations were directly proportional to follicular growth and, as expected, inversely correlated with 8-OHdG levels.

Kang et al. [129] investigated in-vitro porcine oocyte media supplemented with and without melatonin. They found a significantly lower level of ROS and a greater proportion of MII (mature) oocytes in the melatonin group but without an increase in cleavage frequency or blastocyst cell number. Tamura et al. [125] incubated mouse germinal vesicles exposed to H_2_O_2_ with several different concentrations of melatonin. After 12 hours, a positive dose–response relationship was found between increasing amounts of melatonin and the number of mature oocytes. These results strongly suggest that melatonin supplementation in-vitro is associated with a reduction in oxidative stress and improved oocyte maturation.

The literature is conflicting, however, with other animal studies finding an optimal melatonin range of 10^–6^ to 10^–9^ M in in-vitro maturation media, with both higher and lower doses having negative effects [130],[131]. These findings are in agreement with human studies which have demonstrated that lower concentrations of melatonin in culture media improved nuclear maturation rate of immature MI oocytes [132], implantation rate and an insignificant increase in clinical pregnancy rate [133] with an optimal threshold of 10^–5^ M to 10^–9^ M. Both studies agreed that higher concentrations worsened outcomes. Although there is significant evidence to support a role for melatonin in oocyte maturation in-vitro, further investigation is warranted to confirm the optimal effective dose.

A recent review concluded that oral administration of melatonin reduces intrafollicular oxidative damage and increases fertilisation rates [36]. Unfortunately, most studies addressing the use of melatonin in infertility treatment have been conducted with patients as their own controls (`before and after´ comparison) [36],[125],[134]. In the absence of proper control or placebo groups, it must be assumed that any beneficial effects thus observed are explained by the phenomenon of regression toward the mean [135],[136].

Other human studies have been promising, but unfortunately, have also been challenged by design limitations. Eryilmaz et al. [137] performed an unblinded randomise controlled trial assessing melatonin supplementation in women with sleep disturbances undergoing IVF. The investigators randomise 30 patients to receive 3 mg nocte of oral melatonin from day 3–5 of their cycle up until administration of the human chorionic gonadotrophin (HCG) trigger. Controls received no additional treatment. They found a significantly increased number of oocytes, increased number of metaphase II oocytes and increased percentage of Grade 1 embryos (69.3% vs 44.8%, p < 0.05). The authors did not mention controlling or accounting for concurrent adjuvant treatments, nor did they account for the number of previous failed IVF cycles. In addition, their patients had a mean duration of infertility of 6–7 years and the aetiology of infertility was not considered.

Despite its limitations, these findings were in keeping with another larger unblinded randomised trial looking specifically at the effect of melatonin on IVF outcomes. Eighty women were randomised to receiving melatonin 3 mg/day or no treatment from the commencement of GnRH agonist administration. The percentage of mature oocytes was higher in the melatonin group (p < 0.05) as was the proportion of high quality embryos, however, an increase in clinical pregnancy rate did not reach statistical significance [138]. Additionally, patients with cancelled cycles were not included in the analysis making these findings susceptible to attrition bias.

One drawback of the studies already discussed is the lack of a placebo control. Others have overcome the challenge of recruiting patients for a placebo-controlled trial by using adjuvant combinations as a control group. A prospective non-placebo controlled trial comparing myo-inositol (an insulin sensitizing agent) and folate supplementation to myo-inositol, folate and melatonin found that those in the melatonin group achieved a greater number of mature oocytes, fewer immature oocytes and a greater number of top-quality embryos [139]. While this suggests an independent effect of melatonin, it may be that melatonin acts synergistically with these agents, given that melatonin can enhance the effect of other antioxidants (Figure 1). Indeed, other investigators have shown that myo-inositol may also be a useful treatment for infertility in polycystic ovarian syndrome (PCOS), with improvements observed in the quantity of mature oocytes, the number of top quality embryos and the clinical pregnancy rates [140].

#### Effects of melatonin on sperm quality

It appears that the reproductive effects of melatonin do not extend only to the female counterpart, with melatonin receptors being demonstrated on spermatozoa [141]. In general, it is accepted that a higher percentage of motile sperm is associated with improved fertilisation rates and Ortiz et al. has shown that the addition of melatonin to seminal samples can improve the overall motility and the percentage of progressively motile spermatozoa [142],[143]. Melatonin also appears to inhibit apoptosis in spermatozoa, with a reduction in early apoptotic events being demonstrated in human sperm thus prolonging sperm survival [144]. These effects would serve to improve sperm quality, therefore increasing the probability of successful fertilisation.

Melatonin, through its neutralisation of reactive oxygen and nitrogen species, has been shown in both animal and human studies to improve seminal quality in-vitro. A study investigating the addition of melatonin to semen extender in cryopreserved seminal samples from Holstein bulls resulted in amelioration of the oxidative effects of the freeze-thaw process [145]. Studies in rats also have shown that melatonin has a positive effect on sperm that have been subjected to oxidative stress, improving sperm number, viability and motility [146]-[148]. Similar results have been found in a small human study in which in-vitro melatonin-treated samples showed a higher percentage of sperm motility and a lower proportion of non-viable spermatozoa [149]. These authors suggested that the mechanism behind their findings was the result of melatonin neutralising reactive nitrogen species [149].

#### Effects of melatonin on embryo culture media

Following retrieval, the micro-environment that gametes and embryos are cultured in is an essential determinant of subsequent fertilisation and implantation success. Many investigators have studied the impact of melatonin supplementation of in-vitro culture media in porcine, murine and bovine embryo development, overall demonstrating a beneficial effect [150]-[152].

Bovine studies have found a higher cleavage rate, increased 8-cell embryo yield and an increased number of blastocysts and blastocyst hatching in embryos cultured with melatonin concentrations ranging between 10^–5^ and 10^–11^ M [153]-[156]. Like supplementation of oocyte culture media, it appears that higher concentrations of melatonin in embryo culture media can be harmful [157].

Therefore, it appears that in-vitro supplementation of embryo culture media with melatonin has a significant impact on the development and quality of embryos, with lower concentrations being more beneficial (and less harmful) than higher ones.

#### Effects of melatonin on luteal function

Progesterone is an essential hormone in the development of a receptive endometrium and for support of early pregnancy, and without it, pregnancy will fail. In a normal menstrual cycle, this progesterone is provided by the corpus luteum, which develops when the granulosa cells in the ruptured follicle luteinise. A certain level of ROS are required for normal ovulation (follicular rupture) and corpus luteal function. An imbalance of ROS results in oxidative stress and this has been identified as a potential cause of luteal phase defect [158],[159].

Studies have also sought to identify the role of melatonin administration during the luteal phase in patients undergoing IVF. A prospective study of 25 women with luteal phase defect compared 14 women who were given 3 mg/d of melatonin from the time of their HCG trigger throughout the luteal phase with 11 women who were given no supplements. The findings showed that melatonin supplementation significantly increased progesterone levels (11.0 ng/ml vs 8.9 ng/ml, p < 0.05) [160]. Another study found that melatonin can increase serum progesterone levels in women with a luteal phase defect, but this study did not have a control arm and the observed differences in serum concentrations (<10 ng/ml) were not clinically significant, making the relevance of these findings questionable [161]. Consequently, the application of melatonin for luteal phase support is yet to be confirmed.

#### Effects of melatonin on pregnancy rates - human studies

Several trials designed to determine the efficacy of melatonin in improving pregnancy rates have considered it in combination with folic acid and myo-inositol, a B complex vitamin synthesized endogenously from glucose [139],[162]-[164].

Rizzo et al. [139] in a prospective trial of 65 women compared myo-inositol and folate supplementation to myo-inositol, folate and melatonin. They found a trend towards a higher clinical pregnancy rate in the melatonin group, but this did not reach statistical significance.

In a larger double blind randomised controlled trial addressing these agents in PCOS patients undergoing ICSI, Pacchiarotti et al. [164] allocated 178 patients to triple therapy (myo-inositol 4 g, folic acid 400mcg and melatonin 3 mg per day) and 180 patients to myo-inositol and folic acid alone [164]. With this larger sample size, they found higher numbers of mature oocytes (48% vs 35%, p = 0.008) and grade 1 embryos (45.7% vs 30.4%, p = 0.0045) in patients treated with triple therapy, supporting the role of melatonin in the treatment of infertility caused by PCOS. This does not necessarily demonstrate an independent effect of melatonin on embryo quality or oocyte maturity, and as discussed previously, may represent a synergistic effect with myo-inositol and folic acid, although this has not been proven.

Overall, only a limited number of clinical studies have investigated the use of melatonin to improve pregnancy outcomes in infertile women. These studies have generally been poorly designed, have often compared combination regimens, have investigated a narrow range of melatonin doses and have been unable to conclusively identify an independent positive role for melatonin on clinical pregnancy rates after IVF. There clearly is a need for a large randomised double blind placebo-controlled trial to investigate whether oral melatonin increases clinical pregnancy rates in IVF patients and which dose provides maximal benefit.

Tamura et al. [125] investigated the role of melatonin supplementation in 115 patients who failed to become pregnant in a previous cycle of IVF/ET, with a fertilisation rate of less than or equal to 50%. They used a dose of 3 mg/day in the next IVF cycle from day 5 of the menstrual cycle until oocyte retrieval. The fertilisation rate was significantly higher in the melatonin group when compared with their first cycle (50.0 ± 38.0% vs 22.8 ± 19.0%, p < 0.01). In addition, intrafollicular melatonin concentrations were significantly increased and the oxidative stress marker 8-OHdG was significantly decreased by melatonin treatment [125]. Furthermore, the pregnancy rate trended towards an improvement in the melatonin group, albeit not reaching statistical significance.

Another prospective longitudinal cohort study addressed the effects on myo-inositol and melatonin supplementation in women who failed to conceive in previous IVF cycles because of poor oocyte quality [165]. Forty six women were treated with myo-inositol 4 g/day and melatonin 3 mg/day for three months and then underwent another IVF cycle. After this treatment, there were statistically significant improvements in the number of mature oocytes and fertilisation rate. The number of top-quality embryos transferred was also higher than the previous cycle. The clinical pregnancy rate after supplementation was 19.6%. Because this was a before-after study and patients were only included if they failed to conceive in their first cycle, it is difficult to comment on the significance of this clinical pregnancy rate as an appropriate control group was not used.

Unfortunately, both studies were of low quality using a before and after comparison with regression to the mean likely explaining observed differences [136].

#### Systematic reviews and meta-analyses

Only one meta-analysis has been performed specifically assessing the use of melatonin in IVF. This recent systematic review and meta-analysis of five randomise controlled trials found a pooled risk ratio of 1.21 (95% CI 0.98 - 1.50) in favour of melatonin for the outcome of clinical pregnancy rate. However, the authors suggested that the adequacy of the data evaluating the usefulness of melatonin is poor, and that it should not yet be recommended for routine use [166]. While they did not find any worsening of the outcomes of IVF, the authors commented on the lack of live birth rate as an outcome measure as well as the imprecision encountered in all studies considered [166].

On the other hand, melatonin is also known to be remarkably safe, with the Cochrane systematic review and meta-analysis finding no association between antioxidant supplementation and adverse effects for women involved in treatment [88]. This meta-analysis which considered studies of melatonin as well as other antioxidants, found a similar non-statistically significant improvement in clinical pregnancy rate when using any antioxidant (OR 1.30, 95% CI 0.92 - 1.85) with a total sample size of over 2000 patients [88].

### Conclusion and future directions

While the beneficial nature of melatonin, an endogenous anti-oxidant, has been known for decades, the investigation into the role of melatonin in the treatment of infertility is still in its infancy. Good quality evidence has emerged from other disciplines indicating the utility of melatonin in the treatment of a variety of medical conditions. For example, a recent phase II double blind placebo controlled randomised trial has shown that melatonin can help reduce chronic pelvic pain in women with endometriosis potentially through its effects on brain-derived neurotrophic factor and beneficial effects on sleep quality [57]. Level II evidence has also determined the effectiveness of melatonin as an analgesic in temporomandibular disorders [167] and as a method of reducing oxidative stress and improving dyspnoea in patients with chronic obstructive pulmonary disease [6]. Despite this, melatonin use in infertility treatment still lacks adequate evidence to recommend routine use.

Infertility treatments are associated with significant levels of reactive oxygen species which have the potential to negatively affect the quality of oocytes and embryos. Melatonin shows promise as an adjunctive therapy in the treatment of infertility. Its unique anti-oxidative characteristics and safety profile make it an ideal potential adjuvant therapy to be further investigated in well designed double blind randomised placebo-controlled trials.

## Authors’ contributions

SF and LR had significant roles in drafting, revising and authorising this paper for publication. Both authors read and approved the final manuscript.

## Authors’ information

SF (MBBS(hon) BMedSc(hon)) is an Obstetrics and Gynaecology Senior Registrar at Monash Health in Melbourne, Australia. He is also a Clinical Research Fellow and Adjunct Lecturer in the Department of Obstetrics and Gynaecology at Monash University in Melbourne, Australia.

LR is Associate Professor in the Department of Obstetrics and Gynaecology at Monash University and Head of Reproductive Medicine at Monash Medical Centre. He is the Research Director of Monash IVF and a Board Member of the World Endometriosis Society, the World Endometriosis Research Foundation and the Fertility Society of Australia. He also serves on the Grants and Scholarship Committee of the Royal Australian and New Zealand College of Obstetricians and Gynaecologists.

## Authors’ original submitted files for images

Below are the links to the authors’ original submitted files for images. Authors’ original file for figure 1 Authors’ original file for figure 2

## Abbreviations

IVF: In-vitro fertilisation
ART: Assisted reproductive technology
ICSI: Intra-cytoplasmic sperm injection
ET: Embryo transfer
ROS: Reactive oxygen species
DNA: Deoxyribonucleic acid
GnRH: Gonadotrophin releasing hormone
8-OHdG: 8-hydroxy- 2'-deoxyguanosine
MI: Meiosis I
MII: Meiosis II
PCOS: Polycystic ovarian syndrome
HCG: Human chorionic gonadotrophin
LH: Luteinising hormone
