# Supplementary figures and images for: Melatonin: shedding light on infertility? - a review of the recent literature
Source: J Ovarian Res. 2014 Oct 21;7:98. doi: 10.1186/s13048-014-0098-y (PMC4209073; doi:10.1186/s13048-014-0098-y)

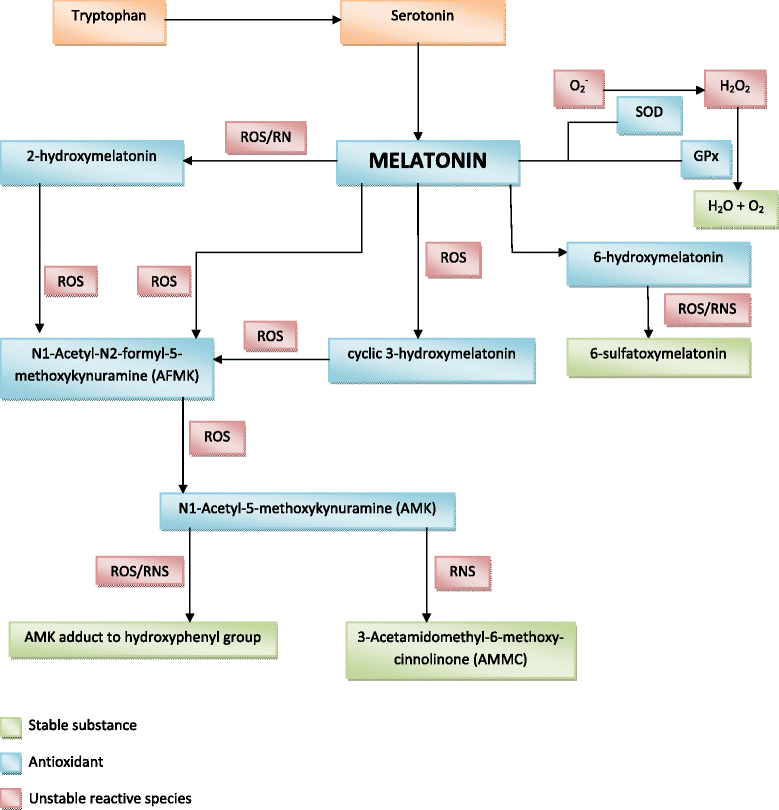

Supplement: Supplementary file 1 — Authors’ original file for figure 1 [file 13048_2014_98_MOESM1_ESM.gif]

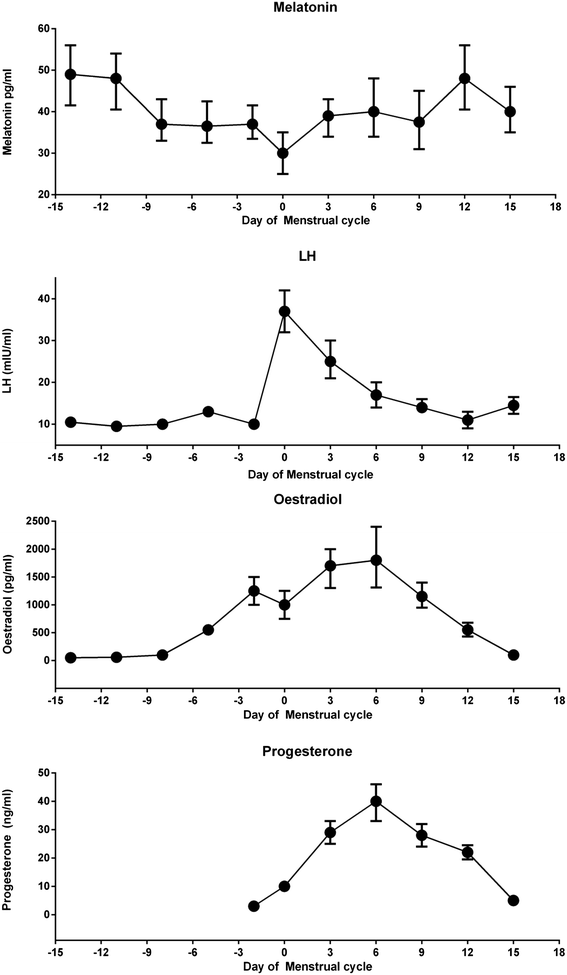

Supplement: Supplementary file 2 — Authors’ original file for figure 2 [file 13048_2014_98_MOESM2_ESM.gif]
